# New evidence in trauma resuscitation - is 1:1:1 the answer?

**DOI:** 10.1186/2047-0525-2-13

**Published:** 2013-07-03

**Authors:** Timothy E Miller

**Affiliations:** 1 Department of Anesthesiology, Duke University Medical Center, 5677 HAFS Building, Box 3094, Durham NC 27710, UK

**Keywords:** Trauma, Resuscitation, Transfusion, Red blood cells, Plasma, Platelets, Massive transfusion protocol, Blood product ratio, Survivor bias

## Abstract

Traumatic injury is a common problem, with over five million worldwide deaths from trauma per year. An estimated 10 to 20% of these deaths are potentially preventable with better control of bleeding. Damage control resuscitation involves early delivery of plasma and platelets as a primary resuscitation approach to minimize trauma-induced coagulopathy. Plasma, red blood cell and platelet ratios of 1:1:1 appear to be the best substitution for fresh whole blood; however, the current literature consists only of survivor bias-prone observational studies.

## Review

### Key points

● Damage control resuscitation is now the predominant focus upon arrival in hospital. Crystalloid delivery should be minimized, as it can be associated with harm.

● Trauma-induced coagulopathy is a common problem caused by dilution and consumption of clotting factors, hypothermia and acidosis. Evidence suggests it can be decreased by early delivery of blood products.

● The best resuscitation appears to be with whole blood rather than component therapy. Can 1:1:1 ever be an adequate substitute for fresh whole blood, or should blood banks consider a return to using whole blood?

● The use of Factor VII is controversial and is usually no longer necessary with early delivery of blood products. It was probably mainly used to treat an iatrogenic coagulopathy. Tranexamic acid should be considered and is significantly cheaper.

● Giving unnecessary plasma and platelets should be discouraged in order to reduce the risk of transfusion-related acute lung injury. Point of care coagulation tests may aid decision-making and reduce unnecessary transfusions.

● Massive transfusion protocols improve communication and delivery of blood products to the patient. They enable clinicians to give fresh plasma up front, rather than giving red blood cells initially and plasma later.

## Review

### The problem of traumatic injury

Traumatic injury is the leading cause of death between the ages of 1 and 40 [1]. An estimated five million people die per year worldwide from traumatic injuries [2]. It has been estimated that 10 to 20% of these deaths are potentially preventable [3]. The biggest cause of preventable death is early hemorrhage within the first 6 hours after incurring an injury [1], which has led trauma teams to investigate whether a change in practice could help reduce early mortality after severe trauma.

It is important to note that only 25% of trauma patients actually receive a blood transfusion, with just 2 to 3% of civilian traumas and 7 to 8% of military trauma patients receiving a massive transfusion (MT) [4]. The definition of a MT varies within the literature. The most commonly used definition is a transfusion of greater than 10 units of packed red blood cells (RBCs) within 24 hours [5].

Patients receiving a MT therefore form a small proportion of the overall trauma burden. The majority of trauma patients do not need blood products or the use of a massive transfusion protocol (MTP). However it is the most severely injured patients requiring MT that are at risk of early hemorrhagic death. These patients have a mortality ranging from 40 to 70% at leading trauma centers [2] and may benefit from the early use of blood products and damage control resuscitation (DCR) as described in this review.

### The history of transfusion

The major changes in blood transfusion practice over the last 100 years have largely occurred based on the experience of military physicians during the major conflicts of the 20th century. The first use of preserved blood for transfusion was carried in 1917 by Captain Oswald H. Robertson from the United States Army Medical Officer Reserves Corps for the British First Army during the Battle of Cambrai in the First World War and was remarkably successful [6,7]. He transfused O-negative blood, which had been stored for 14 days and brought to the front line. This led to the use of transfusion in the final year of the war, in combination with saline and colloids [8]. By the time of the Second World War, albumin and lyophilized (freeze-dried) plasma were being used in combination with whole blood to achieve a balanced resuscitation [9] - not dis-similar to practices that are being advocated today. Since the Second World War we have almost gone full circle, which means that first practice changed for the worse.

Transfusion methods altered dramatically around the time of the Vietnam War in the 1970s, when practice changed from using whole blood to component therapy. This was primarily due to the need for better resource utilization, and to reduce infectious disease transmission. This change in practice was accepted without support from randomized controlled trials or large retrospective studies in MT, and as a rule people did not adapt well to using component therapy. There was confusion as to how to use component therapy for MT, with underuse of clotting products being the norm. As a result, inadvertent hemodilution became routine as a part of MT in the 1970s and 1980s. Failure to control bleeding led to a vicious cycle of problems that became known as the ‘lethal triad’ of trauma: coagulopathy, hypothermia and acidosis.

At that time, giving a significant amount of crystalloid upfront on admission was common practice, which created various problems including abdominal compartment syndrome, acute respiratory distress syndrome (ARDS) and multiple organ failure [10]. Products other than crystalloid were only considered much later in the treatment process after laboratory results had been analyzed or problems had already begun to develop.

Finally, in the late 1990s clinicians started realizing the deleterious effects of excess crystalloid [11,12]. This led to a return to the balanced resuscitation that was reminiscent of that described in the Second World War.

### Trauma-induced coagulopathy

Trauma-induced coagulopathy is an important predictor of blood utilization and trauma-related mortality [13]. It is mainly an iatrogenic or secondary coagulopathy, a condition in which various elements are thought to play a role, including ongoing dilution and consumption of clotting factors from crystalloid and RBCs, acidosis and hypothermia [12].

Recently, it has been proposed that this secondary coagulopathy is preceded by an early trauma-induced coagulopathy (ETIC). ETIC is defined as prolonged PT upon admission and has been described as a separate phenomenon to trauma-induced coagulopathy in that it is an early and primary event. The cause of ETIC is unknown although several theories have been proposed. One theory attempting to explain the cause of ETIC is that the actual injury causes release of tissue factor, which consequently causes thrombin and fibrin generation and utilization, and disseminated intravascular coagulation [14]. Another theory suggests that the hypoperfusion and ischemia known to be associated with trauma can cause release of activated protein C, which leads to consumption of plasminogen activator inhibitor (PAI-1), inhibition of the clotting cascade, systemic anti-coagulation and hyperfibrinolysis [15].

A recent case-control study by Shaz looking only at patients with ETIC found no difference between the cases and the controls in thrombin or fibrin generation, and no difference in the amount of fibrinolysis [16]. It was found that patients with ETIC had been given more crystalloid in the pre-hospital admission phase; therefore they concluded ETIC may not be a unique pathophysiologic response but rather a secondary trauma-induced coagulopathy that occurs before a patient reaches the hospital.

What is clear is that the coagulopathy of trauma (whether it is called ETIC, trauma-induced coagulopathy or alternate terms such early coagulopathy of trauma and acute coagulopathy of trauma shock) is associated with an increase risk of bleeding and mortality [16]. Therefore care must be taken to decrease trauma-induced coagulopathy where possible. This can be achieved by reducing the amount of crystalloids given initially. Ley *et al.* analyzed prospectively collected data from 3,137 trauma patients at a level-1 trauma center between 2000 and 2008 in an attempt to identify the factors responsible for increased mortality after trauma. After multivariate logistic regression analysis the resulting factors included many that were expected including injury severity scores greater than 16, a Glasgow coma scale (GCS) of less than 8, hypotension and an age greater than 80. Another major factor was having received IV crystalloid of greater than 1.5 liters in the emergency department, which was an independent risk factor for mortality in both elderly (>70 years old) (OR 2.89 (1.13 to 7.41, *P* = 0.027) and non-elderly patients ({OR 2.09 (1.31-3.33), *P* = 0.002). High-volume (>3 liter) crystalloid resuscitations were associated with particularly high mortality in elderly patients (OR 8.61 (1.55 to 47.75), *P* = 0.014).

A recent study in South Africa, looking at the use of either colloid or crystalloid in early trauma patients, showed that superior initial resuscitation using colloid instead of crystalloid resulted in decreased lactate levels and less renal injury after penetrating trauma [17]. The superior intravascular retention with the colloid enabled better tissue resuscitation. These results are supported by a recent observational study [18].

### Damage control resuscitation

The concept of damage control resuscitation (DCR) was proposed in the mid 2000s as an alternative resuscitation approach to hemorrhagic shock. Damage control resuscitation involves:

1. Rapid control of surgical bleeding

2. Early and increased use of red blood cells, plasma and platelets in a 1:1:1 ratio

3. Limitation of excessive crystalloid use

4. Prevention and treatment of hypothermia, hypocalcemia and acidosis

5. Hypotensive resuscitation strategies

The first major study looking at damage control resuscitation was carried out by Borgman and colleagues at the US combat hospital in Iraq [19]. This retrospective study looked at 252 patients who had been given a MT, and found a pronounced difference in mortality between patients who had low, medium or high plasma to red blood cell ratios; the higher the plasma ratio in the blood, the lower the risk of mortality. This was achieved by primarily reducing early (<4 hours from admission) death from hemorrhage. Other studies in military trauma have also shown a mortality benefit from high plasma:RBC ratios [20-22].

Holcomb described similar results in 466 MT civilian patients transported to 16 Level 1 trauma centers in the United States [23]. Thirty-day survival was significantly increased in patients with high plasma:RBC ratios (>1:2) compared to those with low plasma:RBC ratios (<1:2). They also showed that a combination of high plasma and high platelet ratios (>1:2) increased 6-hour, 24-hour and 30-day survival. Since studies looking at blood product ratios are trying to prevent death from uncontrolled hemorrhage it is important to measure mortality at 6 hours. Later end-points are also relevant as increased exposure to plasma could be reflected in an increased incidence of late deaths from transfusion related lung injury (TRALI), ARDS and multi-organ failure. Sperry and colleagues found a significant reduction in early (24 hour) mortality with plasma:RBC ratio >1:1.5 in MT, but a higher risk of ARDS [24].

It is important to note that currently all studies looking at blood product ratios in both civilian and military trauma are observational studies. They are therefore prone to survivor bias due to the fact that many deaths occur early in the hospital course when the patients are more likely to be in the low ratio group [25]. A further confounding factor is that patients who have more severe injuries and receive relatively higher quantities of RBCs are both more likely to receive a lower plasma:RBC ratio and are more likely to die [26].

### What about platelets?

After plasma:RBC ratios had been identified as an important component of a MT, Perkins and colleagues focused on platelet ratios in military trauma [27]. The high ratio group received approximately 1:1 platelets to RBCs, and had improved 24-hour and 30-day survival rates compared to other groups. This study however aptly illustrates the problems of survivor bias; the median time to death in the low platelet group was 2.3 hours, whereas the overall median time to administering the first units of platelets was 2.5 hours.

Higher platelet:RBC ratios (>1:2) have also been shown to improve 24-hour and 30-day survival after MT secondary to civilian trauma [23,28,29]. The current US military resuscitation approach is to use 1:1:1 resuscitation for all combat casualties expected to receive a MT [2].

### What about fibrinogen?

One unit of fresh whole blood contains 1000 mg of fibrinogen, therefore losing a unit of blood also dispels 1000 mg of fibrinogen. It is common practice to replace this loss with one unit of RBCs and one unit of fresh frozen plasma (FFP), which restores approximately 500 mg of fibrinogen, and so in a MT it is necessary to add more fibrinogen at a later stage. This is usually done with cryoprecipitate, which is derived from the precipitate fraction of cold-thawed human plasma. For example, 10 units of cryoprecipitate given in a MT will contain 2.5 g of cryoprecipitate, thus compensating for the fibrinogen deficit.

Stinger *et al.* showed that a high fibrinogen:RBC ratio (>0.2 g of fibrinogen:RBC) was independently associated with survival to discharge after military trauma (survival 76% versus 48%, *P* <0.001) [20]. As 1 unit of cryoprecipitate contains 0.25 g of fibrinogen that ratio can be achieved by transfusing cryoprecipitate:RBC in a 1:1 ratio. In practice this is usually done by transfusing one 10-unit bag of cryoprecipitate for every 10 units of red cells transfused.

A cryoprecipitate:RBC ratio of 1:1 has also been shown to reduce 24-hour and 30-day mortality after MT in civilian trauma [28].

### The advantages of warm fresh whole blood

Spinella *et al.* compared the use of warm fresh whole blood (WFWB) to component therapy from a transfusion database at the US Army Institute of Surgical Research [21]. They looked at all combat casualty patients from both Iraq and Afghanistan who were transfused with >1 unit of RBCs. The patients who received WFWB got on average only 30% WFWB and 70% component therapy, however their survival rate was far better than patients who had received component therapy alone.

The results of this study suggest that there must be a significant advantage to using WFWB over component therapy. Patients receiving WFWB are given 500 ml of warm blood with no storage deficits and good hematocrit levels. WFWB also contains the full amount of platelets, clotting factors and fibrinogen and is therefore healthier and more beneficial to the patient. Even ‘best’ practice component therapy in a 1:1:1 ratio of platelets, plasma and RBCs does not contain comparable levels of platelets, clotting factors, or fibrinogen [30]. When components are reconstituted after the addition of anti-coagulants and additive solutions a cold, dilute product is produced. Storage lesions also occur in the stored blood products leading to decreased RBC deformability and reduced platelet function [30,31]. Therefore even best practice component therapy using the 1:1:1 formula is not as effective as fresh whole blood.

### Other drug therapies

The use of recombinant Factor VII (rFVII) in trauma is controversial, and has decreased in recent years. It may be that rFVII was used to treat an iatrogenic resuscitation injury caused by overuse of crystalloid and RBCs. As a practice has developed of giving more plasma and platelets upfront, rFVII has fallen out of use and is frequently no longer necessary in trauma surgery. One study investigating the use of Factor VII within a major trauma center found no correlation between the use of the drug and outcome benefit; its use seemed to merely prolong death rather than prevent it [32]. Other trials in have also shown a lack of benefit [33] and evidence of harm [34].

Tranexamic acid has been suggested as a cheaper alternative to Factor VII. The Crash-2 trial randomized over 20,000 trauma patients to either tranexamic acid or control. Tranexamic acid significantly reduced the risk of death (OR 0.91 (0.85 to 0.97), *P* = 0.0035) and death from hemorrhage (0.85 (0.76 to 0.96), *P* <0.001), without any increase in thromboembolic complications [35]. The drug should therefore be considered in all patients requiring MT. However, it should be noted that the treatment produced only marginally superior outcomes compared to the control, and only reached significance due to the large number of patients studied.

### Survivor bias

There is a methodological issue with the literature on MT, as no randomized control trial exists on increased blood product ratios. Observational studies on transfusion ratios are prone to survivor bias (SB) as many of the deaths occur early in the hospital course when administration of plasma typically lags behind RBCs, and they therefore fall into the low plasma:RBC ratio group. Conversely patients surviving long enough to receive sufficient plasma fall into the high plasma:RBC group. In order to avoid SB some investigators exclude patients who die within the first hour or two after arriving at the Emergency Department. Alternatively investigators can model the relationship between mortality and plasma:RBC ratio over time, and treat the ratio as a time-dependent covariate.

Ho *et al.* recently reviewed the entirety of the literature on blood product ratios in trauma to examine the prevalence of survivor bias [25]. They reviewed 26 studies on blood ratios in trauma and found that:

● 11 studies that showed a benefit in high plasma:RBC ratios were SB-prone. This included all four military studies

● 10 studies that showed a benefit in high plasma:RBC ratios were considered SB-unlikely

● 5 other studies were SB-unlikely but showed no benefit in high plasma:RBC ratios.

Therefore while SB might partly explain the large reduction in mortality with high plasma:RBC ratios in some studies, there is still a considerable body of evidence in favor of early administration of blood products within a MTP.

### Massive transfusion protocols

The improvement in mortality with changes in blood product ratios has led many hospitals to implement MTPs [36]. MTPs vary between institutions, but the principles remain the same. MTP activation is usually at the discretion of the trauma surgeon or emergency department physician. Upon activation, blood services will deliver several ‘rounds’ of blood products containing RBCs, plasma and platelets to the patient until the protocol is deactivated.

Riskin and colleagues showed that deaths from trauma significantly decreased after the introduction of a MTP [37]. They were already practicing an aggressive transfusion practice in an attempt to prevent dilutional coagulopathy, and the survival benefit does not appear to have been related to any alteration in the volume or ratio of blood components used. However by allowing expeditious product availability, the MTP resulted in earlier transfusion of RBCs and a significant decrease in time to first transfusion of plasma and platelets.

The key to the success of the protocol was therefore improved communication and organization within the MTP enabling earlier delivery of blood products from blood services. A MTP should enable clinicians to administer 1:1 plasma to RBCs immediately upon admission to hospital, rather than giving RBCs initially and then plasma at a later stage. This means that thawed plasma has to be available in the emergency department for use in the first round of the MTP instead of having to wait for FFP.

Thawed plasma is simply FFP, which after thawing, is kept at 4°C for 5 days. Thawed AB plasma is stored in emergency department refrigerators alongside emergency release type O blood. This allows both products to be used immediately and concurrently when a MTP is initiated.

MTPs improve patient survival and also reduce clinician stress. Another advantage is that MTPs have the potential for cost savings with earlier control of hemorrhage leading to decreased overall blood products utilization. O’Keefe and colleagues reported a saving of $2,270 per patients after introduction of a MTP [38].

### How can we predict who needs a massive transfusion protocol?

It is extremely important to predict who will need a MT so that the MTP can be implemented to help prevent early hemorrhagic deaths. However only 2% of civilian trauma patients need a MT, so that means that the vast majority of trauma patients do not need a MTP implemented. This cannot be overstated as overuse of a MTP can cause harm as well as leading to wastage in blood bank products and resources. There is a significant and well-established risk of TRALI when giving blood components (predominantly plasma). There is currently no data in trauma, but in non-trauma studies plasma transfusion was associated with an almost threefold increased risk of acute lung injury (ALI) (OR 2.92 (1.99 to 4.29), *P* = 0.14) [39]. The proposed mechanism of TRALI involves antibodies from donor blood components that are directed against Human Leucocyte Antigens (HLA). These antibodies are most numerous in women who have been pregnant, hence the risk of TRALI can be reduced by using predominantly male plasma [40].

It is sometimes difficult to predict who will need a MTP activated. Dente and colleagues reported an ‘overtriage’ rate of 27% in whom the protocol was activated but the patient never received a MT [41]. This led them to look for early clinical markers after torso gunshot wounds which correlated with the need for MT. They noted that all patients with transpelvic and mulitcavity gunshot wounds required MT. Of the patients with isolated transabdominal or transthoracic bullet trajectories, many did not require MT. However a systolic pressure less than 90 mmHg and a base deficit of greater than −10 units were strong predictors of the need for MT.

Ultimately a universal set of activation triggers may not be possible due to practice variations at different trauma centers, and the final decision will rely on the experience and judgment of the trauma team.

### Point of care coagulation testing

Point of care (POC) coagulation testing is an attractive alternative to formula-driven approaches to blood transfusion in trauma. POC hemoglobin, prothrombin time, platelet count and fibrinogen level and thromboelastography (TEG™ Haemonetics Corp., Braintree, MA, USA) and thromboelastometry (ROTEM™, Tem International GmbH, Munich, Germany) are all available. Currently POC testing is more commonly used in the majority of trauma patients who have significant injuries but not enough to activate a MTP, although as improvements occur in the speed and accuracy of these technologies they may become an alternative to formula-driven MTPs in the most severe trauma.

Whole-blood viscoelastic tests, such as TEG or ROTEM provide a real-time graphic representation of clot formation enabling clinicians to individualize correction of coagulopathy more accurately and substantially faster than standard coagulation tests [42]. There is increasing evidence that these devices are helpful in guiding coagulation therapy for trauma patients according to their actual needs. Individualized coagulation management can potentially reduce the risks or both under-transfusion and over-transfusion of blood products [43]. However POC coagulation testing is not yet a standard of care in most hospitals, and studies are needed to demonstrate improved patient survival or decreased use of blood products.

### Future directions

Several large-scale trials are ongoing that may aid clinicians in the management of MT. The US Department of Defense-sponsored PROMMTT prospective observational study, which was performed at 10 major U.S. trauma centers, has recently been completed and will produce a lot of data on the management of MT in major centers and outcomes [44].

The first multi-center randomized controlled trial to compare blood ratios in trauma is being planned in the United States. The PROPPR (Prospective Randomized Optimum Platelet and Plasma Ratios) trial will enroll 580 patients in 12 centers over 2 years (ClinicalTrials.gov Identifier: NCT01545232) Patients will be randomized to either 1:1:1 or 1:1:2 (platelets:plasma:RBCs),

A formula versus lab-guided study comparing 1:1 versus conventional resuscitation has recently been completed (ClinicalTrials.gov Identifier: NCT00945542), and a trial to look at the use of stored whole blood in civilian trauma is currently in progress. (ClinicalTrials.gov Identifier: NCT01227005).

### So where are we now?

The current available evidence suggests that in severe trauma with hemorrhagic shock a MTP should be activated. Patients should be resuscitated with warm fresh whole blood or best practice component therapy in a ratio of 1:1:1:1 (plasma:platelets:cryoprecipitate:RBCs). Tranexamic acid should also be considered. In other words, if the patient is hemorrhaging whole blood we should reconstruct whole blood as much as we can. The benefit of preventing an early hemorrhagic death far outweighs the risk of subsequent TRALI.

Some authors are advocating a return to the widespread use of WFWB in major trauma centers [45]. Transfusion of WFWB has been associated with improved survival during MT, and also substantially reduces recipient exposure to plasma and platelets, thereby reducing the risk of TRALI. However, there are major logistical difficulties in supplying WFWB that would need to be overcome to overhaul blood services.

Once there is control of surgical bleeding in MT, and for all patients who did not need a MT point of care coagulation tests should be used wherever possible. If there is no imminent danger of life threatening hemorrhage if seems prudent to use POC tests to ‘fine tune’ use of blood products and prevent unnecessary administration.

## Conclusions

Reduced crystalloid use upfront, massive transfusion protocols and 1:1:1 in the absence of whole blood have all been shown to improve trauma outcomes. There is growing evidence to suggest that 1:1:1 is the best possible alternative to fresh blood, together with damage control resuscitation. However the principles of damage control resuscitation should only be applied to those patients who are going to need massive transfusion with life-threatening hemorrhage.

Once surgical control has been achieved, and in the 98% of patients who do not require a massive transfusion, the ultimate goal would be to individualize blood product ratios from patient to patient rather than having a set ratio for all cases. This could be achieved using point of care coagulation tests, allowing for the delivery of RBCs, plasma and platelets as and when the patient needs them.

It is likely that there will be a significant amount of literature in the near future on the use of lyophilized plasma and other blood products, which will be of particular advantage in smaller hospitals and in combat zones where disease-free blood products will be immediately available. There may also be an increased drive toward more widespread use of warm fresh whole blood. Future clinical trials will define the role of these products and continue the advances in trauma care that have been made over the last decade.

## Abbreviations

ALI: Acute lung injury; ARDS: Acute respiratory distress syndrome; DCR: Damage control resuscitation; ETIC: Early trauma-induced coagulopathy; rFVII: Recombinant factor seven; FFP: Fresh frozen plasma; GCS: Glasgow coma score; HLA: Human leucocyte antigens; MT: Massive transfusion; MTP: Massive transfusion protocol; PAI-1: Plasminogen activator inhibitor; POC: Point of care; RBC: Red blood cell; SB: Survivor bias; TRALI: Transfusion-related acute lung injury; WFWB: Warm fresh whole blood.

## Competing interests

The author has received honoraria from Hospira, Covidien, and Edwards Lifesciences, and received research funding from Cheetah Medical and Retia Medical.
